# The Prevention of Gonorrhœa

**Published:** 1898-04

**Authors:** 


### THE PREVENTION OF GONORRHŒA.

A. Neisser recommends the method proposed by Blodasewski. It consists in instilling (not injecting) a few drops of a two per cent. solution of nitrate of silver into the meatus after coitus, a drop also being allowed to flow over the frænum. Experiments have shown that a two per cent. solution of silver nitrate kills the gonococcus.—*Med. and Surg. Reporter.*

